# Genetic Influences on Exercise Participation in 37.051 Twin Pairs from Seven Countries

**DOI:** 10.1371/journal.pone.0000022

**Published:** 2006-12-20

**Authors:** Janine H. Stubbe, Dorret I. Boomsma, Jacqueline M. Vink, Belinda K. Cornes, Nicholas G. Martin, Axel Skytthe, Kirsten O. Kyvik, Richard J. Rose, Urho M. Kujala, Jaakko Kaprio, Jennifer R. Harris, Nancy L. Pedersen, Janice Hunkin, Tim D. Spector, Eco J.C. de Geus

**Affiliations:** 1 Department of Biological Psychology, Vrije Universiteit Amsterdam, Netherlands; 2 Queensland Institute of Medical Research Brisbane, Australia; 3 Epidemiology, Institute of Public Health, University of Southern Denmark Odense, Denmark; 4 Department of Psychology, Indiana University Bloomington, Indiana, United States of America; 5 Department of Health Sciences, University of Jyäskylä Jyäskylä, Finland; 6 Department of Public Health, University of Helsinki Helsinki, Finland; 7 Department of Mental Health and Alcohol Research, National Public Health Institute Helsinki, Finland; 8 Norwegian Institute of Public Health Oslo, Norway; 9 Department of Medical Epidemiology and Biostatistics, Karolinska Institutet Stockholm, Sweden; 10 Twin Research and Genetic Epidemiology Unit, St Thomas' Hospital London, United Kingdom; University of California, San Francisco, United States of America

## Abstract

**Background:**

A sedentary lifestyle remains a major threat to health in contemporary societies. To get more insight in the relative contribution of genetic and environmental influences on individual differences in exercise participation, twin samples from seven countries participating in the GenomEUtwin project were used.

**Methodology:**

Self-reported data on leisure time exercise behavior from Australia, Denmark, Finland, Norway, the Netherlands, Sweden and United Kingdom were used to create a comparable index of exercise participation in each country (60 minutes weekly at a minimum intensity of four metabolic equivalents).

**Principal Findings:**

Modest geographical variation in exercise participation was revealed in 85,198 subjects, aged 19–40 years. Modeling of monozygotic and dizygotic twin resemblance showed that genetic effects play an important role in explaining individual differences in exercise participation in each country. Shared environmental effects played no role except for Norwegian males. Heritability of exercise participation in males and females was similar and ranged from 48% to 71% (excluding Norwegian males).

**Conclusions:**

Genetic variation is important in individual exercise behavior and may involve genes influencing the acute mood effects of exercise, high exercise ability, high weight loss ability, and personality. This collaborative study suggests that attempts to find genes influencing exercise participation can pool exercise data across multiple countries and different instruments.

## Introduction

Regular exercisers have reduced cardiovascular morbidity and mortality [1]–[3]. In addition, exercisers are characterized by enhanced psychological well-being and sharper minds. They have a lower incidence of depression and anxiety disorders [4]–[7] and show cognitive advantages, specifically in frontal executive functions [8]–[11]. These advantages for mental and physical health are well-known. Even so, a large part of the population remains nearly completely sedentary [12]–[14] and this percentage appears to be resistant to more than 50 years of population campaigning. As a consequence, a sedentary lifestyle remains a major threat to health in contemporary societies.

Studies on the determinants of exercise behavior have mainly focused on social and environmental characteristics like access to facilities [15], [16], socioeconomic status [13], [16], race [17], job strain [18], [19], marital status [17], subjective “lack of time” [20], [21], health beliefs [13], and social support by family, peers or colleagues [21]–[23]. Despite their face validity, none of these factors has emerged as a strong causal determinant of exercise behavior [24], [25]. Increasingly, therefore, the influence of biological factors has been considered [26]–[28].

Dispositional differences in the drive to exercise will be most obvious in leisure time, i.e. self-chosen, exercise behavior. Parent-offspring studies have confirmed a significant familial influence on leisure time exercise participation [29]–[32] and twin studies have further shown this influence to reflect the shared genetic make-up of family members [33]–[41]. The estimates of genetic contribution are very inconsistent, ranging from no genetic effects [30] to a high heritability [33]. These inconsistencies may reflect relatively small samples sizes and different definitions of exercise participation. They may also reflect a change in genetic architecture with age, or true socio cultural differences in the relative contribution of the environment related to country-specific traditions, attitudes about exercise, and opportunities to engage in exercise [13].

In this paper, we estimated the heritability of exercise participation using very large twin samples from seven countries participating in the GenomEUtwin project, a multinational collaboration of twin registries aiming to uncover the genetic variation that influences, amongst others, risk factors for cardiovascular disease.

## Methods

### Study population

This study is based on (repeated) surveys in twin samples from seven countries participating in the GenomEUtwin project: Australia, Denmark, Finland, the Netherlands, Norway, Sweden, and United Kingdom. The exact descriptions of the twin registries of these countries have been described in detail elsewhere [41]–[44]. We restricted our analyses to adults aged 19 to 40 years.

When exercise data were available from more than one survey in a country, we used the most recent survey. If only one twin had completed the most recent survey, we searched for the most recent survey that was completed by both members of the pair. If the other member never filled out a survey, the single twin was nonetheless retained in the analysis to improve on the estimation of exercise prevalence and its variance. Only complete twin pairs, however, are informative for the analyses of twin resemblance. Below, the surveys from which the data are drawn are briefly described by country. The final sample sizes are summarized in Table 1.

**Table 1:**
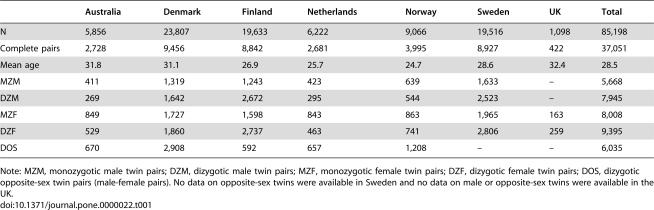
Number of twins in the countries participating in the GenomEUtwin project.

	Australia	Denmark	Finland	Netherlands	Norway	Sweden	UK	Total
N	5,856	23,807	19,633	6,222	9,066	19,516	1,098	85,198
Complete pairs	2,728	9,456	8,842	2,681	3,995	8,927	422	37,051
Mean age	31.8	31.1	26.9	25.7	24.7	28.6	32.4	28.5
MZM	411	1,319	1,243	423	639	1,633	–	5,668
DZM	269	1,642	2,672	295	544	2,523	–	7,945
MZF	849	1,727	1,598	843	863	1,965	163	8,008
DZF	529	1,860	2,737	463	741	2,806	259	9,395
DOS	670	2,908	592	657	1,208	–	–	6,035

Note: MZM, monozygotic male twin pairs; DZM, dizygotic male twin pairs; MZF, monozygotic female twin pairs; DZF, dizygotic female twin pairs; DOS, dizygotic opposite-sex twin pairs (male-female pairs). No data on opposite-sex twins were available in Sweden and no data on male or opposite-sex twins were available in the UK.

#### Australia

Data were obtained from two different mail surveys conducted in 1980 and 1990. Combining the data from the two surveys and selecting twin pairs between the ages of 19 and 40 years, gave a total of 5,856 participants and 2,728 complete twin pairs.

#### Denmark

Data were derived from three different mail surveys conducted in 1995, 1997 up to 2000, and 2002. The final sample consisted of 23,807 participants and 9,456 complete twin pairs between ages 19 and 40 years.

#### Finland

The Finnish data were obtained from two different mail surveys. The first survey, of the older Finnish Twin Cohort, was conducted in 1975 and consists of same-sex twins born before 1958 [35]. The second survey is from participants in *FinnTwin16*, which consists of twins born in 1975–1979. Data were collected at four time points from an age 16 baseline (ages 16, 17, 18½, and 22–25). For these analyses, we used survey data from the fourth wave assessment when twins were between the ages of 23 to 27 years [45]. Combining the two cohorts and selecting the 19 to 40 year old twins resulted in a total of 19,633 participants and 8,842 complete twin pairs.

#### The Netherlands

The Dutch data were obtained from a longitudinal study on health and lifestyle in twin families registered with the Netherlands Twin Registry (NTR). Since 1991, every two to three years, twins and their families have received a mail survey [46]. Combining the six surveys (1991, 1993, 1995, 1997, 2000, and 2002) and excluding the twins younger than 19 and older than 40, resulted in a total sample of 6,222 participants and 2,681 complete twin pairs.

#### Norway

The Norwegian exercise data were derived from two mail surveys, the first in 1992, and the second in 1998 [47]. Combining the two surveys and excluding the twin pairs younger than 19 years resulted in a total sample of 9,066 participants and 3,995 complete twin pairs.

#### Sweden

The Swedish data were obtained from a mail survey sent in 1972 to all same-sex twin pairs born in 1926–1958 [48]. The final sample includes a total of 19,516 participants older than 18 years and younger than 41 years of which 8,927 complete twin pairs could be formed.

#### United Kingdom

Exercise data from two studies in the St. Thomas' UK Adult Twin Registry (TwinsUK) were used for the analyses. The first study assessed self-reported exercise behavior with a detailed mail survey on health and lifestyle sent out in 2000. The second study comprises data from clinical interviews on lifestyle that were held between 1992 and 2001. Some twins participated in one interview, while others have been interviewed twice. Exercise data from the two studies were combined and the pairs older than 40 and younger than 19 years were removed from the data set. As numbers of male pairs in the age range were small, only female data were retained, resulting in a total sample of 1,098 female participants, from which 422 complete twin pairs could be formed.

### Exercise participation

Different exercise questions were asked in each of the countries. Systematic coding of duration, frequency and intensity was not possible in all countries. We therefore aimed to define a dichotomy that would be reasonably comparable across countries. Subjects were classified as exercisers if they met a predefined criterion that corresponded to about 60 minutes of weekly exercise activities with a minimum intensity of four metabolic equivalents (METs), where one MET is the rate of energy expenditure of an individual sitting quietly, which is approximately one kcal/kg/h. They were classified as non-exercisers otherwise.

In Australia, to meet the criterion, subjects had to exercise in their leisure time once a week with a minimal intensity comparable to moderate activities like gardening; in Denmark, they had to engage in hard physical activity (contrasted with light physical activity) outside their working hours for at least one hour a week; in Finland, they had to engage in leisure time exercise at least once a week with a minimum intensity comparable to light jogging for a duration of at least one hour; in the Netherlands they had to engage in one or more leisure time exercise activities with a minimum intensity of four METs, and the total time spent on all such activities was at least 60 minutes a week; in Norway, they exercised during leisure time between one and two times a week at sufficient intensity to build up a sweat and with each session between 30–60 minutes in duration; in Sweden, they had to exercise “rather a lot”, “a lot” or “really a lot” (in contrast to “not very much”, “rather little”, “very little”, and “almost none”); in the UK, they had to be regularly engaged in exercise activities with a minimum intensity of four METs.

### Analysis of twin similarity

#### Correlations

Comparing the correlations of MZ and DZ twins provides information about the nature of the influences contributing to the twin resemblance. MZ twins are genetically identical, while DZ twins share on average half of their segregating genes. If MZ twins resemble each other more than DZ twins, this is an indication that genetic factors (A) play an important role in explaining individual differences in exercise participation. Similar MZ and DZ twin correlations suggest that common environmental factors (C), i.e. factors shared by members of a twin pair, influence variance in exercise participation, because the common environment is similar in MZ and DZ twins [49]. Finally, an MZ intrapair correlation different from unity suggests unique environmental effects (E), i.e. factors not shared by members of a twin pair plus measurement error, because MZ twins have identical common environments and identical genes. Adding dizygotic opposite-sex twins (DOS) to the twin design enables us to investigate sex differences. If the DOS correlation is lower than the same-sex dizygotic correlations, this indicates that different common environmental or genetic effects influence exercise participation in males and females.

#### Threshold model

We estimated tetrachoric correlations from a standard liability threshold model [50]. The model assumes that there is an underlying liability for exercise behavior, which is continuous and normally distributed in the population. This underlying normal distribution is divided by a threshold, which is obtained from the observed proportions of exercisers and non-exercisers. Individuals whose scores fall below the threshold, which can be interpreted as a *z*-value, do not meet the exercise criteria and are classified as non-exercisers; those with scores exceeding the threshold are classified as regular exercisers. The thresholds may, or may not be equivalent for males and females, which will be tested.

#### Model fitting procedure

We used structural equation model (SEM) fitting to partition the variance in latent liability into three components, i.e. genetic, common environmental, and unique environmental factors. The basic principles of structural equation modeling of twin data have been outlined elsewhere [49]. A detailed treatise on the statistical testing procedure is found in Neale & Cardon [51]. Different models were fitted to raw ordinal data using the software package Mx [51]. First, we fitted a saturated model to estimate the tetrachoric correlations between twins. The saturated model is fully parameterized (i.e. it has no constraints) and is used to evaluate the fit of nested, more restricted models. If the fit of a nested model is significantly worsened (*p*<0.01), the predicted contributions of genetic and environmental factors are inconsistent with the data, and the nested model should be rejected. Alpha levels were set to .01 in all samples.

Using nested models, we tested whether the prevalence of exercise was the same for males and females, whether there was an effect of age on the prevalence of exercise, and whether this effect was the same for both sexes. Next, we tested whether different genes in males and females contribute to the liability to exercise participation, and whether the magnitude of the contribution of genes and environment was the same in males and females. Finally, we analyzed whether both genetic and common environmental factors play a role in familial resemblance by consecutively constraining their contribution to exercise participation to zero. In each country, the most parsimonious model was retained to estimate the relative contribution of genes, common environment shared by family members, and unique environment to individual differences in exercise participation.

## Results

**Figure 1:**
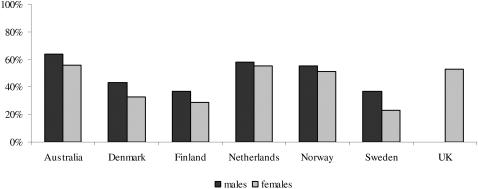
Prevalence of exercise participation by country and sex.

Prevalence of exercise participation for the seven countries is given in Figure 1, which shows that the percentage of male exercisers is generally higher than the percentage of female exercisers. The average percentage of male and female exercisers was 44% and 35% respectively. Lowest participation was found in Sweden (37% for males and 23% for females) and highest participation in Australia (64% for males and 56% for females).

Exercise prevalence remained stable across this age range only for males and females in the Netherlands and for females in the UK and Sweden. The prevalence of exercise gradually decreased from age 19 to age 40 in the other countries and the decrease with age in prevalence was the same for males and females.

For all zygosity groups in the different countries, Table 2 displays the tetrachoric correlations. The resemblance in exercise participation of MZ twin pairs was higher as that for DZ twins, consistent with a genetic influence on exercise participation. With the exception of Finland, the DOS correlations were significantly lower than the dizygotic same-sex correlations. This indicates that the genetic factors influencing exercise participation in males do not completely overlap with those in females.

**Table 2:**
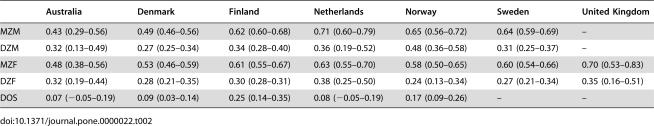
Twin correlations and 95% CI intervals (between parentheses) for exercise participation by country and zygosity group.

	Australia	Denmark	Finland	Netherlands	Norway	Sweden	United Kingdom
MZM	0.43 (0.29–0.56)	0.49 (0.46–0.56)	0.62 (0.60–0.68)	0.71 (0.60–0.79)	0.65 (0.56–0.72)	0.64 (0.59–0.69)	–
DZM	0.32 (0.13–0.49)	0.27 (0.25–0.34)	0.34 (0.28–0.40)	0.36 (0.19–0.52)	0.48 (0.36–0.58)	0.31 (0.25–0.37)	–
MZF	0.48 (0.38–0.56)	0.53 (0.46–0.59)	0.61 (0.55–0.67)	0.63 (0.55–0.70)	0.58 (0.50–0.65)	0.60 (0.54–0.66)	0.70 (0.53–0.83)
DZF	0.32 (0.19–0.44)	0.28 (0.21–0.35)	0.30 (0.28–0.31)	0.38 (0.25–0.50)	0.24 (0.13–0.34)	0.27 (0.21–0.34)	0.35 (0.16–0.51)
DOS	0.07 (−0.05–0.19)	0.09 (0.03–0.14)	0.25 (0.14–0.35)	0.08 (−0.05–0.19)	0.17 (0.09–0.26)	–	–

Table 3 shows the relative contribution of genetic influences (A) to the total variance in exercise behavior in each country, also known as its heritability. In addition, the relative contribution of common (C) and unique environmental (E) influences are given. Sequential model fitting (depicted in Table 4) suggested that the contribution of additive genetic and unique environmental factors to the variance in exercise participation was significant in all samples, but that common environmental factors only contributed significantly to exercise participation of the Norwegian males.

**Table 3:**
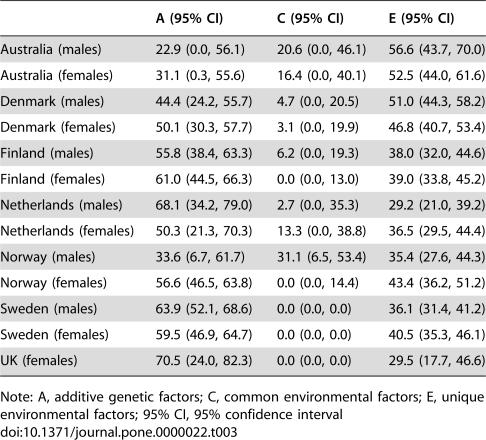
Heritability estimates and confidence intervals by country for the full model.

	A (95% CI)	C (95% CI)	E (95% CI)
Australia (males)	22.9 (0.0, 56.1)	20.6 (0.0, 46.1)	56.6 (43.7, 70.0)
Australia (females)	31.1 (0.3, 55.6)	16.4 (0.0, 40.1)	52.5 (44.0, 61.6)
Denmark (males)	44.4 (24.2, 55.7)	4.7 (0.0, 20.5)	51.0 (44.3, 58.2)
Denmark (females)	50.1 (30.3, 57.7)	3.1 (0.0, 19.9)	46.8 (40.7, 53.4)
Finland (males)	55.8 (38.4, 63.3)	6.2 (0.0, 19.3)	38.0 (32.0, 44.6)
Finland (females)	61.0 (44.5, 66.3)	0.0 (0.0, 13.0)	39.0 (33.8, 45.2)
Netherlands (males)	68.1 (34.2, 79.0)	2.7 (0.0, 35.3)	29.2 (21.0, 39.2)
Netherlands (females)	50.3 (21.3, 70.3)	13.3 (0.0, 38.8)	36.5 (29.5, 44.4)
Norway (males)	33.6 (6.7, 61.7)	31.1 (6.5, 53.4)	35.4 (27.6, 44.3)
Norway (females)	56.6 (46.5, 63.8)	0.0 (0.0, 14.4)	43.4 (36.2, 51.2)
Sweden (males)	63.9 (52.1, 68.6)	0.0 (0.0, 0.0)	36.1 (31.4, 41.2)
Sweden (females)	59.5 (46.9, 64.7)	0.0 (0.0, 0.0)	40.5 (35.3, 46.1)
UK (females)	70.5 (24.0, 82.3)	0.0 (0.0, 0.0)	29.5 (17.7, 46.6)

Note: A, additive genetic factors; C, common environmental factors; E, unique environmental factors; 95% CI, 95% confidence interval

**Table 4:**
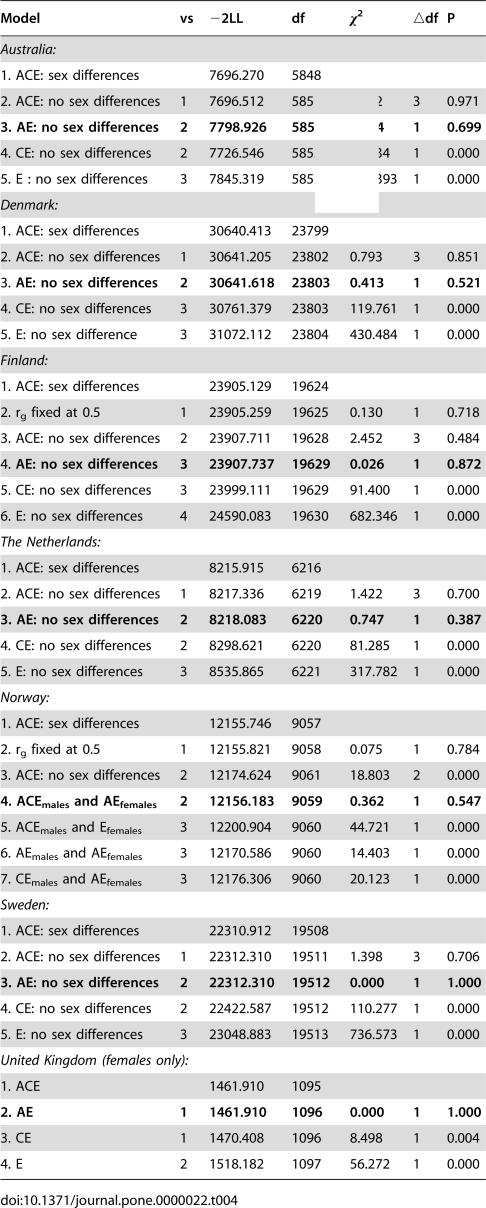
Univariate model fitting results for twins in the 7 different countries; comparisons of models are shown.

Model	vs	−2LL	df	χ^2^	▵df	P
*Australia:*						
1. ACE: sex differences		7696.270	5848			
2. ACE: no sex differences	1	7696.512	5851	0.242	3	0.971
**3. AE: no sex differences**	**2**	**7798.926**	**5852**	**2.414**	**1**	**0.699**
4. CE: no sex differences	2	7726.546	5852	30.034	1	0.000
5. E : no sex differences	3	7845.319	5853	146.393	1	0.000
*Denmark:*						
1. ACE: sex differences		30640.413	23799			
2. ACE: no sex differences	1	30641.205	23802	0.793	3	0.851
3. **AE: no sex differences**	**2**	**30641.618**	**23803**	**0.413**	**1**	**0.521**
4. CE: no sex differences	3	30761.379	23803	119.761	1	0.000
5. E: no sex difference	3	31072.112	23804	430.484	1	0.000
*Finland:*						
1. ACE: sex differences		23905.129	19624			
2. r_g_ fixed at 0.5	1	23905.259	19625	0.130	1	0.718
3. ACE: no sex differences	2	23907.711	19628	2.452	3	0.484
4. **AE: no sex differences**	**3**	**23907.737**	**19629**	**0.026**	**1**	**0.872**
5. CE: no sex differences	3	23999.111	19629	91.400	1	0.000
6. E: no sex differences	4	24590.083	19630	682.346	1	0.000
*The Netherlands:*						
1. ACE: sex differences		8215.915	6216			
2. ACE: no sex differences	1	8217.336	6219	1.422	3	0.700
**3. AE: no sex differences**	**2**	**8218.083**	**6220**	**0.747**	**1**	**0.387**
4. CE: no sex differences	2	8298.621	6220	81.285	1	0.000
5. E: no sex differences	3	8535.865	6221	317.782	1	0.000
*Norway:*						
1. ACE: sex differences		12155.746	9057			
2. r_g_ fixed at 0.5	1	12155.821	9058	0.075	1	0.784
3. ACE: no sex differences	2	12174.624	9061	18.803	2	0.000
**4. ACE_males_ and AE_females_**	**2**	**12156.183**	**9059**	**0.362**	**1**	**0.547**
5. ACE_males_ and E_females_	3	12200.904	9060	44.721	1	0.000
6. AE_males_ and AE_females_	3	12170.586	9060	14.403	1	0.000
7. CE_males_ and AE_females_	3	12176.306	9060	20.123	1	0.000
*Sweden:*						
1. ACE: sex differences		22310.912	19508			
2. ACE: no sex differences	1	22312.310	19511	1.398	3	0.706
**3. AE: no sex differences**	**2**	**22312.310**	**19512**	**0.000**	**1**	**1.000**
4. CE: no sex differences	2	22422.587	19512	110.277	1	0.000
5. E: no sex differences	3	23048.883	19513	736.573	1	0.000
*United Kingdom (females only):*						
1. ACE		1461.910	1095			
**2. AE**	**1**	**1461.910**	**1096**	**0.000**	**1**	**1.000**
3. CE	1	1470.408	1096	8.498	1	0.004
4. E	2	1518.182	1097	56.272	1	0.000

Heritability estimates and confidence intervals under the best fitting models in each country are shown in Table 5. Heritability of exercise participation in males ranged from 27% in Norway to 67% in the Netherlands and in females from 48% in Australia to 71% in the UK. The median figure for all groups was 62%.

**Table 5:**
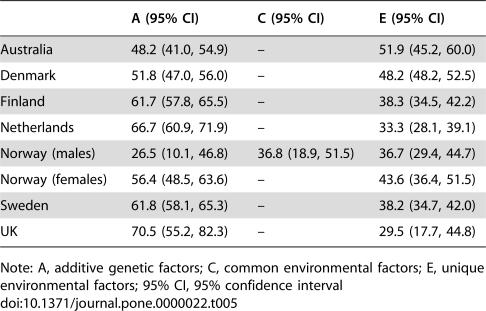
Heritability estimates and confidence intervals by country for the most parsimonious model.

	A (95% CI)	C (95% CI)	E (95% CI)
Australia	48.2 (41.0, 54.9)	–	51.9 (45.2, 60.0)
Denmark	51.8 (47.0, 56.0)	–	48.2 (48.2, 52.5)
Finland	61.7 (57.8, 65.5)	–	38.3 (34.5, 42.2)
Netherlands	66.7 (60.9, 71.9)	–	33.3 (28.1, 39.1)
Norway (males)	26.5 (10.1, 46.8)	36.8 (18.9, 51.5)	36.7 (29.4, 44.7)
Norway (females)	56.4 (48.5, 63.6)	–	43.6 (36.4, 51.5)
Sweden	61.8 (58.1, 65.3)	–	38.2 (34.7, 42.0)
UK	70.5 (55.2, 82.3)	–	29.5 (17.7, 44.8)

Note: A, additive genetic factors; C, common environmental factors; E, unique environmental factors; 95% CI, 95% confidence interval

## Discussion

This study compared the intrapair resemblance in exercise behavior in 13,676 MZ twin pairs to that in 23,375 DZ twin pairs from seven different countries. In all countries, a significant contribution of genetic factors to exercise participation in leisure time was found. The median heritability of exercise participation was 62% across the seven countries and ranged from 27% in Norwegian males to 70% in female twins from the UK. These findings underscore the robustness of the genetic contribution to this lifestyle behavior. Different birth cohorts and survey periods were studied across the countries and different questions were used to assess regular exercise in each of the countries. Moreover some countries used clinical interviews as well as mail questionnaires. Despite this variation in the assessment instruments and the inclusion of different age cohorts, highly comparable results were found in all countries, as evidenced by the substantial overlap in the heritability estimates. Common environmental factors shared by the twins in their youth such as home environment, school and peer group attitudes and behavior appear to play only a modest role in adult exercise behavior (with the exception of the Norwegian males).

What is the nature of the genetic factors causing individual differences in voluntary exercise behavior? In part, such factors may act through personality, which has been shown to be heritable almost without exception [52]. Conscientiousness, self-motivation, and self-discipline are essential to adhere to a chosen long term goal even if it violates immediate needs and such factors have long been implied as important determinants of exercise behavior [24]. Neuroticism, anxiety, and depression are all associated with lower exercise prevalence [4], [53]. This association has been explained as reflecting a causal effect of exercise, but reversed causality cannot be ruled out. Low self-esteem and depressed mood may well act against participation in exercise, particularly when this needs to be done in an evaluative context.

Individual differences in nervous system structure and function that are related to personality may also influence the degree to which the act of exercising itself is rewarding to some and aversive to others. The immediate aversive effects caused by exercise-related fatigue related to monoamine depletion [54] may depend on genetic differences in monoaminergic systems. The extent of immediate rewarding effects may well depend on genetic variation in the opioid and dopamine systems [55]. Genetic differences in aversive/rewarding effects may also be found in the period after exercise. For instance, strong cardiac vagal control enabling faster heart rate recovery, a genetically influenced trait [56], may tip the balance between rewarding and aversive effects of acute exercise in favor of reward, by reducing some of the aversive effects of exercise (e.g. prolonged palpitations). Likewise, the temporary reduction in sympathetic stress reactivity after exercise [57] and the positive mood states paired to it [58] may depend on the exact genotype of the subjects.

Finally, there are powerful social-psychological mechanisms that may make some people more attracted to exercise than others. Given the strong positive cultural attitudes towards exercise ability, people who notice that they are better in exercise than others will experience stronger feelings of competence and mastery and may find it easier to adhere to regular exercise. Both endurance and strength traits have been shown to be highly heritable [59]–[61]. Genes that favor basal physical fitness or the responsiveness to training programs, therefore, may also predispose to exercise behavior. A second related mechanism may be genetic differences in body composition and specifically the ability to lose weight in response to exercise [62]. The desire to lose weight is a frequently cited reason for participation in exercise across many different countries [13]. Hence, a genetic advantage in the ability to lose weight through exercise may facilitate adherence to regular exercise.

The latter two mechanisms may also explain why different genes were found to influence exercise participation in males and females (significantly so in the Australian, Danish and Dutch samples). This may reflect a sex difference in the relative subjective importance of exercise ability and exercise-induced weight loss. Among adolescents, for instance, the most commonly reported benefit of exercising for females is “to stay in shape”, whereas the most commonly reported benefit of exercising among males is “to become strong” [63]. Genes favoring fitness may be more relevant to male exercise participation, whereas genes favoring weight loss may have a larger impact on female participation.

Our threshold models detected modest geographical variation in exercise participation. We hesitate to interpret these prevalence differences as meaningful, because different and imperfect instruments were used to query exercise in the seven countries. Self-reported exercise shows only imperfect correlation to more objective measures like energy expenditure obtained from double water labeling methods or actometer recordings [64]. Furthermore, from the surveys used we could not sufficiently determine duration and intensity of exercise activities in each of the countries to obtain a quantitative measure like METhours per week. Resorting to a dichotomy clearly limited the validity of our exercise measure, particularly when comparing prevalences across countries. A further limitation was the difference in the birth cohorts. Data in Finland and Sweden were collected in the late 70's, which is on average more than 15 years earlier than data collection in the other countries. Analyses of the secular trends in Finland and Sweden showed that more people are currently engaging in regular exercise in these countries than was the case in the seventies.

In spite of these limitations, Figure 1 does not seem to paint an encouraging picture of the exercise habits in the seven participating countries. Even at our mild criterion of about 60 minutes at four METs weekly, only about 50% of the subjects were classified as being regularly active in leisure time across all seven countries. This low prevalence of regular leisure time exercise has been a cause for concern in many countries, and encouragement of a more active lifestyle is an important component of international public health recommendations [65]. Identification of the genetic factors that underlie the significant heritability of exercise participation may improve our understanding of why some people fail to engage in regular exercise and potentially improve our ability to intervene. For exercise *ability*, coordinated efforts are ongoing worldwide and a number of genes for endurance and strength have been identified and replicated [60], [66]. For exercise *behavior*, no such coordinated effort exists. Here we show that such efforts could successfully pool databases of genotype and exercise information across multiple countries to enhance detection of the genomic regions implied in exercise behavior.
